# Hox cluster characterization of Banna caecilian (*Ichthyophis bannanicus*) provides hints for slow evolution of its genome

**DOI:** 10.1186/s12864-015-1684-0

**Published:** 2015-06-18

**Authors:** Riga Wu, Qingfeng Liu, Shaoquan Meng, Peng Zhang, Dan Liang

**Affiliations:** State Key Laboratory of Biocontrol, Key Laboratory of Gene Engineering of the Ministry of Education, School of Life Sciences, Sun Yat-Sen University, Guangzhou, 510275 People’s Republic of China; College of Life Science and Technology, Yulin Normal University, Yulin, 537000 People’s Republic of China

## Abstract

**Background:**

Caecilians, with a discrete lifestyle, are the least explored group of amphibians. Though with distinct traits, many aspects of their biology are poorly investigated. Obtaining the caecilian genomic sequences will offer new perspectives and aid the fundamental studies in caecilian biology. The caecilian genomic sequences are also important and practical in the comparative genomics of amphibians. Currently, however, only sparse genomic sequences of caecilians are available. Hox genes, an old family of transcription factors playing central roles in the establishment of metazoan body plan. Understanding their structure and genomic organization may provide insights into the animal’s genome, which is valuable for animals without a sequenced genome.

**Results:**

We sequenced and characterized the Hox clusters of Banna caecilian (*Ichthyophis bannanicus*) with a strategy combining long range PCR and genome walking. We obtained the majority of the four caecilian Hox clusters and identified 39 Hox genes, 5 microRNA genes and 1 pseudogene (*ψHoxD12*). There remained seven intergenic gaps we were unable to fill. From the obtained sequences, the caecilian Hox clusters contained less repetitive sequences and more conserved noncoding elements (CNEs) than the frog counterparts. We found that caecilian and coelacanth shared many more CNEs than frog and coelacanth did. Relative rate of sequence evolution showed that caecilian Hox genes evolved significantly more slowly than the other tetrapod species used in this study and were comparable to the slowly evolving coelacanth Hox genes. Phylogenetic tree of the four Hox clusters also revealed shorter branch length especially for the caecilian HoxA, HoxB and HoxD clusters. These features of the caecilian Hox clusters suggested a slowly evolving genome, which was supported by further analysis of a large orthologous protein dataset.

**Conclusions:**

Our analyses greatly extended the knowledge about the caecilian Hox clusters from previous PCR surveys. From the obtained Hox sequences and the orthologous protein dataset, the caecilian Hox loci and its genome appear evolving comparatively slowly. As the basal lineage of amphibians and land vertebrate, this characteristic of the caecilian genome is valuable in the study concerning the genome biology and evolution of amphibians and early tetrapods.

**Electronic supplementary material:**

The online version of this article (doi:10.1186/s12864-015-1684-0) contains supplementary material, which is available to authorized users.

## Background

Caecilians (Gymnophiona) together with frogs (Anura) and salamanders (Caudata) constitute the three living orders of the Class Amphibia. Caecilians live in the tropical regions of southeast Asia, Africa, the Seychelles islands and Central and South America. They primarily have a terrestrial, surface-cryptic or burrowing lifestyle as adults. Because of their secretive habits, caecilians are not frequently observed in the wild and are relatively unknown. There are currently 199 recognized caecilian species, which is far fewer than the numbers of salamanders (659) and frogs (6367) [1]. Although the group is small, caecilians are intriguing to scientists because they have many traits that readily distinguish them from frogs and salamanders. For example, consistent with their fossorial lifestyle, caecilians’ body is greatly elongated and segmented by annular grooves [2]. They also lack sternal elements, girdles and limbs, and their tail is either very short or absent. These characteristics make them look like snakes. However, as a non-model cryptic organism, many aspects of caecilian’s biology are poorly investigated. Phylogenetically, caecilians are placed in a key position on the vertebrate tree. They are the basal lineage of modern amphibians [3–6], and are therefore the most primitive group of land vertebrates. The genomic information of caecilian will not only aid the biological studies on these animals but also will be important in addressing issues concerning the evolution of early land vertebrates, such as the water-to-land transition. Currently, however, only a sparse number of caecilian nucleotide sequences are available in public databases, and most of them are mitochondrial genes from phylogenetic studies. Obtaining more genomic information on caecilians is urgently important.

Hox genes are usually among the first genes investigated in a non-model organism. They are an old family of transcription factors characterized by a highly conserved 180 bp motif, the homeobox [7]. They govern the timing and route of segmental development along the bilaterian animal body axis and are also involved in the patterning of the limbs and organogenesis [8–10]. In vertebrates, Hox genes are organized into several clusters that exhibit colinearity between the gene order in the cluster and the temporal and spatial expression order of the Hox genes during embryonic development: genes at the 3′ ends of the clusters are expressed earlier in development and more anterior than genes at the 5′ ends of the clusters [11, 12]. This colinearity is thought to be regulated by the many cis-regulator elements within and outside the Hox clusters. Thus, both the Hox gene coding sequences and the noncoding regions in the Hox clusters are important in maintaining proper Hox functions. As a result of their significant involvement in early development, changes in expression of Hox genes may cause severe morphological and/or physiological alterations, which are expected to play important roles in driving evolutionary changes.

Hox genes have been extensively studied in a variety of animals, not only for their importance during development, but also for their genomic organization, such as number of genes, number of clusters and elements in the non-coding regions. Often, the genomic organization of Hox clusters provides insights about the animal’s genome. For example, a pattern from one single Hox cluster in the cephalochordate amphioxus [13] to four paralogous Hox clusters (termed HoxA, HoxB, HoxC and HoxD) in tetrapods was one of the empirical findings that support the two round genome duplication hypothesis [14]. The subsequent discovery of extra Hox clusters (up to 7 or more clusters) in teleost fishes was the first indication of an additional teleost-specific genome duplication [15, 16]. The comparatively slow evolution of the Hox clusters in coelacanth was among some of the earliest molecular evidence supporting the slow evolution of its genome [17, 18]. In anole lizard, a massive enrichment of transposable elements at the Hox loci was revealed, reflecting a genomic character that a large number of transposable elements accumulated in the development-related-gene-containing regions [19]. Thus, information on Hox clusters is valuable, especially for animals of which the genome has not been sequenced.

For the cryptic caecilians, we and other labs have used comprehensive PCR surveys of the conserved homeodomains to make inferences on the number of Hox genes [20, 21]. Sequencing of the posterior fragment of the HoxD cluster (from *Evx2* to *HoxD10*) in the caecilian *Typhlonectes natans* was recently achieved based on a BAC library and revealed an expansion and multiple repeats in the intergenic region between *HoxD13* and *HoxD11* [22]. To better characterize the caecilian Hox clusters and better understand the caecilian genome, we aimed to sequence the four Hox clusters of the Banna caecilian. With no access to a BAC library, we used the results from our previous PCR survey and an alternative strategy that combined long-range PCR and genome walking. We successfully obtained the majority of the Hox cluster sequences, which greatly increased our knowledge of the caecilian Hox clusters. Based on these findings, we determined most of the Hox genes’ genomic linkages; screened for repetitive sequences, identified conserved non-coding elements using a comparative genomics approach, and measured the relative rates of evolution of the caecilian sequences in comparison with those of other sarcopterygians. In our obtained caecilian Hox cluster sequences, there were fewer repetitive sequences and more conserved noncoding elements than found in the frog homolog sequences, and the caecilian Hox genes appear to be evolving relatively slowly. This information from the caecilian Hox clusters hinted at a slowly-evolved caecilian genome and we further tested it using a large dataset of 623 orthologous protein genes.

## Methods

### Sequencing and annotation of the Banna caecilian Hox clusters

This study was performed in strict accordance with the guidelines developed by the China Council on Animal Care and Use. All animal processing procedures were approved by the Institutional Animal Care and Use Committee of Sun Yat-Sen University (permit number: 2011-023). Genomic DNA was extracted from ethanol-preserved tissues (liver or muscle) of Banna caecilian (*Ichthyophis bannanicus*; collected in the Guangxi province, China) using the standard salt extraction protocol. We amplified fragments of the Hox clusters with a combination of LA PCR and GW. Primers with high annealing temperature (65–68 °C, 28–35 bp) that were suitable for LA PCR and GW were designed in various anchors, including the Banna caecilian Hox coding regions and the highly conserved noncoding regions within the Hox clusters. The highly conserved noncoding regions were identified based on the multiz alignments of human, mouse, chicken, anole lizard, frog and zebrafish Hox clusters constructed at the UCSC Genome Bioinformatics [23]. LA PCR was conducted to amplify the regions between every two adjacent anchors using TaKaRa LA Taq DNA polymerase (Takara, Dalian) according to standard protocol. If there were weak or no bands on the agarose gel electrophoresis after the first-round of LA PCR, nested PCR amplification was performed. For regions that still failed to amplify, GW was conducted using a Genome Walking Kit based on the TAIL-PCR technique (Takara, Dalian) to shorten the distance between the two adjacent anchors. The physical linkages between the GW acquired fragments and the anchors were further verified by PCR amplification with primers designed in the anchors and the distal ends of the GW acquired fragments. After verification, the GW acquired fragments served as new anchors in which new primers were designed, and LA-PCR was performed again with the new primers to amplify the regions. Finally, we obtained 29 LA PCR fragments and a number of GW fragments.

Fragments less than 5 kb long were sequenced on the ABI3730XL sequencing platform. Longer fragments (≥5 kb) were sequenced using Ion Torrent at Life Technologies Corporation. To prepare the sample, long PCR products (≥5 kb) were pooled in proportion to their length and digested with NEB Next ds DNA fragmentase according to the manufacturer’s instructions to generate a sequencing library with 300–600 bp DNA fragments. Sequence reads obtained from Ion PGM were *de novo* assembled using the MIRA program (version 3.4.1.1) [24] in its ‘genome’ assembly type with quality grades set as ‘accurate’. Together, the assembled contigs from MIRA and sequence fragments of 39 Banna caecilian Hox genes were reassembled using SeqMan in DNASTAR Lasergene software (version 7.1.0) and manually edited. The ambiguous contigs were verified by both blastn search against Indonesian coelacanth or human Hox clusters and further PCR surveys. The remaining gaps were filled through PCR amplification and primer walking.

To annotate the newly obtained caecilian Hox cluster sequences, we used GenomeScan [25] and blastx search against Indonesian coelacanth or human Hox clusters to identify the Hox coding sequences. Exon-intron boundaries were determined manually based on the consensus splice motifs.

### Screening of repetitive elements

Identification and classification of repetitive elements was conducted using Censor [26] with the default parameters against the Repbase library of vertebrate repeat sequences as well as using RepeatMasker 4.0.5 [27]. Simple repeats and low-complexity sequences were excluded. In addition to our newly obtained caecilian Hox cluster sequences, genomic sequences of the four Hox clusters in human (*Homo sapiens*), tammar wallaby (*Macropus eugenii*), chicken (*Gallus gallus*), anole lizard (*Anolis carolinensis*), Western clawed frog (*Xenopus tropicalis*), Indonesian coelacanth (*Latimeria menadoensis*), spotted gar (*Lepisosteus oculatus*), and elephant shark (*Callorhinchus milii*) were extracted from the Ensembl [28] and NCBI nucleotide databases [29] for comparison. When comparing the repeat content among the human, wallaby, chicken, lizard, frog, caecilian and coelacanth Hox clusters, only regions homologous to the obtained Hox sequences in caecilian were analyzed, and regions homologous to the gaps in caecilian were excluded. And “density of repetitive sequences”, which was calculated as the ratio of the total length of repetitive sequences to the length of the clusters in each species, was used. When comparing the distribution of the repetitive sequences in caecilian and frog, all the available Hox cluster sequences of the two amphibians were analyzed.

### Identification and analyses of CNEs

Hox clusters of human, tammar wallaby, chicken, anole lizard, Western clawed frog, Banna caecilian, Indonesian coelacanth, spotted gar and elephant shark were aligned using the global alignment program LAGAN [30] available on the VISTA website [31] and screened for the presence of CNEs. Because of the poor sequence coverage (less than half of the cluster), the chicken HoxC cluster was not included in the analysis. Banna caecilian and the Western clawed frog were used as reference sequence, respectively. The CNEs were restricted to the sequences with a cut-off of ≥65 % identity across windows ≥50 bp. To determine the evolutionary origin of the CNEs, all the CNEs were classified into 4 phylogenetic groups, “gnathostome”, “osteichthyan”, “sarcopterygian” and “tetrapod”. The “gnathostome” group was defined as CNEs conserved in the elephant shark and caecilian (using caecilian as the reference) or frog (using frog as the reference). The “osteichthyan” group referred to CNEs shared by the spotted gar and caecilian or frog. The “sarcopterygian” group referred to CNEs found in coelacanth and caecilian or frog. The remaining CNEs were all classified in the “tetrapod” group. And CNEs already included in one phylogenetic group were not counted in the next group.

### Relative rate test of gene evolution

To determine the rate of evolution of caecilian relative to other species, we performed the Tajima relative rate test [32] on the Hox family. First, each gene-set was separately aligned using muscle 3.6 [33] and ambiguous alignment regions were removed using Gblocks (version 0.91b) [34] with no gaps allowed. Sites with gaps or unknown data were excluded from the RRT. Each comparison included two ingroups and one outgroup. The RRT on each gene-set was performed using in-house python scripts. For most of the Hox genes, the RRTs were performed using Banna caecilian, Western clawed frog, Puerto Rican worm lizard (*Amphisbaena caeca*) (our unpublished data)/Chinese softshell turtle and human as the ingroups and Indonesian coelacanth as the outgroup. The RRTs on *HoxC1* were conducted with elephant shark as the outgroup and caecilian, African lungfish, eel and zebrafish as the ingroups because the Western clawed frog, Puerto Rican worm lizard and human do not have *HoxC1* gene. For *HoxC3*, the ingroups were changed to caecilian, frog, Alpine stream salamander and African lungfish and the outgroup was Indonesian coelacanth. Multiple RRTs can be combined to determine the relative evolutionary speeds of several species. A Hasse diagram, in which the slower-evolving genes were placed below the faster-evolving ones, was used to show the combined RRT results. The significance test corresponding to each RRT was drawn as a solid line (*p* < 0.01) or a dotted line (0.01 < *p* < 0.05). When comparing the rates between caecilian and coelacanth, Elephant shark was used as the outgroup. We also performed an RRT on a carefully curated dataset consisting of 623 orthologous genes, in which caecilian, frog and human were used as the ingroups and coelacanth was used as the outgroup. For both Hox family and the 623 orthologous genes, both nucleotide sequences and protein sequences were analyzed.

### Phylogenetic analysis

Phylogenetic trees were constructed using Hox cluster sequences and the 623 orthologous genes. The four Hox clusters from various gnathostomes were aligned with LAGAN. And the 623 orthologous genes were aligned with muscle 3.6. The ambiguous positions in each alignment were eliminated by Gblocks (version 0.91b) with half gaps allowed. Maximum likelihood trees were constructed with RAxML (version 7.2.6) [35] under the GTR + GAMMA + I model for nucleotide sequences and the LG + GAMMA model for protein sequences. Node support was estimated from 500 rapid bootstrap replicates.

## Results

### Hox clusters in Banna caecilian

**Fig. 1:**
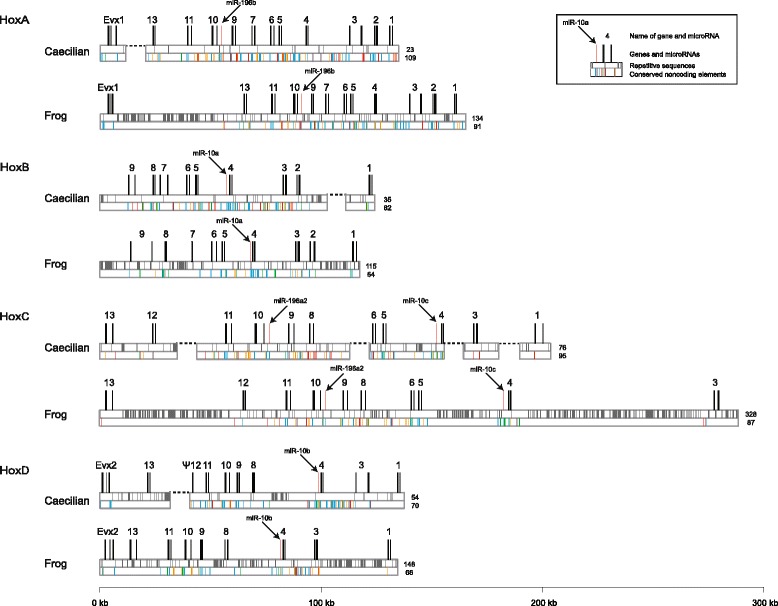
Genomic organization of the Hox clusters in Banna caecilian and Western clawed frog. Schematic representations of the HoxA, HoxB, HoxC and HoxD clusters in caecilian are shown, with the frog Hox clusters drawn for comparison. For each Hox cluster, location of the genes, repetitive sequences and conserved noncoding elements are separately illustrated from *top* to *bottom*. Total numbers of the repetitive sequences and conserved noncoding elements are indicated at the end of each cluster. Exons of the genes are characterized by *black bars* and pseudogenes are denoted by the symbol *Ψ*. Gaps in the intergenic regions are indicated by *dotted lines*. Repetitive sequences identified by Censor are represented by *grey bars*. Conserved noncoding sequences predicted using mVISTA are classified into 4 phylogenetic groups (see Methods for details): “gnathostome (*blue bars*)”, “osteichthyan (*green bars*)”, “sarcopterygian (*red bars*)” and “tetrapod (*orange bars*)”. For clarity, CNEs conserved in caecilian and frog only are not shown in the figure

Based on the Hox gene fragments from our previous PCR survey, we used a strategy that combined long-range PCR (LA PCR) and genome walking (GW) to determine the sequences of the four Hox clusters in Banna caecilian. Primers were designed in the coding regions of Banna caecilian Hox genes and the highly conserved noncoding regions in the sarcopterygian Hox cluster alignments. After assembly, all the physical linkages between the newly acquired fragments were determined, which were then verified by further PCR amplification. In total, we retrieved 527.7 kb sequences of Banna caecilian Hox clusters, which included the nearly complete HoxA cluster (*Evx1* with flanking regions and *HoxA13*-*HoxA1*; 126.9 kb), most of the HoxB cluster (*HoxB9*-*HoxB2* and*HoxB1*with flanking regions; 115.3 kb), part of the HoxC cluster (*HoxC13*-*HoxC12*, *HoxC11*-*HoxC8*, *HoxC6*-*HoxC4*, *HoxC3* with flanking regions and *HoxC1* with flanking regions; 168 kb) and most of the HoxD cluster (*Evx2*-*HoxD13* and *HoxD11*-*HoxD1*; 128.8 kb) (see Fig. 1). The NCBI accession numbers of the four Hox clusters are from KF787115 to KF787118. We were unable to sequence seven intergenic regions most likely because of the limitations of our strategy. They were mainly located in the HoxC cluster and the extremities of the HoxA, HoxB and HoxD clusters. Four of these regions (*Evx1*-*HoxA13*, *HoxC4*-*HoxC3*, *HoxC3*-*HoxC1* and *HoxD13*-*HoxD11*) were putatively very large as estimated by previous studies in other animals [17, 19], and therefore, beyond the scope of long range PCR. The remaining three gaps (*HoxB2*-*HoxB1*, *HoxC12*-*HoxC11* and *HoxC8*-*HoxC6*) may have been due to either enlarged gene distances or complex structures that hindered the progression of the Taq DNA polymerase.

Estimated from the sequences we obtained, the sizes of the caecilian Hox A, HoxB and HoxD clusters are comparable to their counterparts in another amphibian, the Western clawed frog. In both species, the HoxA (*HoxA13*-*HoxA1*), HoxB (*HoxB9*-*HoxB2*) and HoxD clusters (*HoxD11*-*HoxD1*) are ~100, ~80 and ~95 kb in size, respectively. For the less complete HoxC cluster, three fragments were compared. The *HoxC11*-*HoxC8* and *HoxC6*-*HoxC4* fragments in the two species were ~40 kb in size; while the *HoxC13*-*HoxC12* fragment in caecilian (22.8 kb) was approximately only one-third of the size of its counterpart in frog (63.4 kb). For the HoxA, HoxB and HoxD clusters, gene content and gene organization were largely determined. For HoxC cluster, gene linkages were determined except for *HoxC12*-*HoxC11*, *HoxC8*-*HoxC6*, *HoxC4*-*HoxC3* and *HoxC3*-*HoxC1*. The gene content and organization of the caecilian Hox clusters were similar to those in other amphibians, with slight differences. Caecilian retained *HoxC1*, which was lost in many other vertebrates, including frog and salamander, but detected in coelacanth and lungfish. The complete coding sequence of *HoxC1* that we obtained demonstrates that the caecilian *HoxC1* is fully functional. *HoxD12* was completely absent in frog and salamander, but remnants of the caecilian *HoxD12* was observed in region 5′ to *HoxD11* after performing a global alignment with other gnathostomes (Additional file 1).

In our caecilian Hox cluster sequences, we found five microRNA genes, two belong to family *mir-196* and three belong to family *mir-10*, at the same genomic location observed in other vertebrates. *miRNA 196a-1* which is usually located approximately 6 kb 5′ to *HoxB9* in other vertebrates was not identified even though 12.9 kb of sequence 5′ to the caecilian *HoxB9* was screened (see the VISTA plot of *HoxB9* and the 5′ region in Additional file 2). We could not detect *miRNA 196a-1* in the corresponding genomic region in Western claw frog, either. Thus, the *miRNA 196a-1* microRNA in amphibians may be either lost or located further away from *HoxB9*.

In summary, we found in Banna caecilian 39 Hox genes (including *HoxC1* which is not found in frog) and 1 pseudogene (*ψHoxD12*) organized into four clusters, 2 Evx paralogs associated with the HoxA (putatively) and HoxD clusters, respectively, and 5 microRNAs (*miRNA196a-1* yet unidentified).

### Distribution of repetitive sequences

**Fig. 2:**
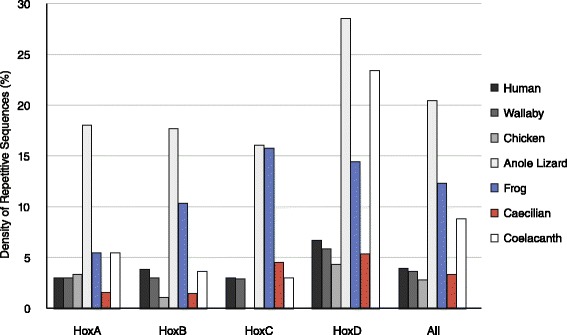
Densities of repetitive sequences of the gnathostome Hox clusters. Density of repetitive sequences for a specific Hox cluster was calculated by dividing the total length of the repetitive sequences by the total length of the cluster. Only repetitive sequences identified by Censor with default settings were calculated. The chicken HoxC cluster was not included in the analysis because it is currently missing many sequences

Obtaining the majority of the caecilian Hox clusters allowed us to investigate the details of the Hox cluster structure and elements in the intergenic regions. First, we evaluated the repetitive sequences which are usually strongly excluded from vertebrate Hox clusters. We used Censor and the Repbase database to detect repetitive sequences in the obtained Banna caecilian Hox clusters and the corresponding regions in the human, wallaby, chicken, lizard, frog and coelacanth Hox clusters. The density of repetitive sequences (calculated by dividing the total length of repetitive sequences by the length of the clusters analyzed) in the caecilian Hox clusters was 3.3 %, among the lowest densities of all the species studied (Fig. 2). It is possible that the density of repetitive sequences in Banna caecilian may have been underestimated without a specific repeat library available. Wallaby, lizard and coelacanth, however, do not have specific repeat libraries either, and they did not show a general bias towards low densities of repetitive sequences; and all of these species had a higher density of repetitive sequences than Banna caecilian. Thus, the effect of databases in identifying repeats does not appear to be predominant and the low density of repetitive sequences was considered primarily as a characteristic of the caecilian Hox cluster sequences that we obtained. When the two amphibians were compared, the repeat content of the Hox clusters in caecilian was approximately only a quarter of that in frog (3.3 % vs. 12.3 %). To avoid any algorithm bias, RepeatMasker was also used to detect repetitive sequences. The density of repetitive sequences in the caecilian Hox clusters (2.3 %) was again among the lowest densities and much lower than that of frog (5.5 %). Because the general trend was consistent in the two analyses, only the results of Censor are shown for the following comparisons.

The attributes and distribution of the repeats detected in the caecilian and frog Hox cluster sequences (including the intergenic regions with gaps) were further investigated (see Fig. 1). Note that because all the unsequenced gaps might contain repeats, the number of repeats in each caecilian Hox cluster was likely underestimated. The most abundant type of repeats in caecilian was non-LTR retrotransposons (46.3 %; the common ones were SINE, L1 and CR1), while in frog approximately 70.3 % of the repeats were DNA transposons, such as hATs, Harbingers and Koloboks. Among the four Hox clusters, the caecilian HoxA cluster contained the least number of repetitive sequences (23 repeats) interspersed in the cluster; the frog HoxA cluster contained 134 repeats, 59 % of which were concentrated in the *Evx1*-*HoxA13* region. In the caecilian HoxB cluster, 35 repeats were found, and over half of them were located between *HoxB2* and *HoxB1*; while in the frog HoxB cluster, 115 repeats were identified and a large portion of them were located in regions 5′ to *HoxB9*, *HoxB8*-*HoxB6* and *HoxB2*-*HoxB1*. For both species, the HoxC cluster contained the most abundant amount of repetitive sequences, with 76 and 328 repeats in caecilian and frog, respectively. While the caecilian repeats were largely interspersed in the cluster, 86.3 % of the frog repeats were concentrated in the two terminal regions, i.e., upstream of *HoxC12* and downstream of *HoxC5*. For the HoxD cluster, 49.6 % of the 54 repeats in caecilian were located in the *HoxD13*-*HoxD11* and *HoxD8*-*HoxD4* regions, while approximately 50 % of the 148 repeats in frog were in the *HoxD3*-*HoxD1* region. Thus, the distribution patterns of the repeats had “hotspots” that were in different locations in the two amphibians. And most of these “hotspots” were enlarged compared with the orthologous regions in other vertebrates. Two of the gapped intergenic regions in caecilian, *HoxB2*-*HoxB1* and *HoxD13*-*HoxD11*, were among the repeat “hotspots”. They might be also enlarged, thus were hard to amplify.

To identify direct and inverted repeats that were not in the known repeat libraries, self-self blastn analyses (minimum identity of 70 %, e-value = 10^−5^) were performed. A direct/inverted repeat consists of two repeat copies (hereafter termed arms) that are approximately identical/complementary to each other. This analysis revealed the presence of 29 inverted repeats and 45 direct repeats in the obtained caecilian Hox clusters, over half of which were identified in the HoxD cluster. In particular, we found two long inverted repeats in the *HoxD8*-*HoxD4* intergenic region. The arms of these two repeats were both longer than 100 bp (125 and 231 bp), and the arm identity was almost 100 %. The spacers between the two arms of both repeats were small (8 and 16 bp). In frog, 78 inverted repeats and 197 direct repeats were found in the self-self blastn analysis in corresponding regions of the Hox clusters, but no long inverted repeat was identified. Indeed, inverted repeats with an arm size longer than 100 bp are rare in genomes. There are only 134 such repeats found in the human genome [36], and currently, no long inverted repeat has ever been reported in the Hox clusters of other gnathostomes species. Determining whether there is any functional significance of these two long inverted repeats in the caecilian Hox cluster is worthy of further investigation.

### Conserved noncoding elements in caecilian Hox clusters

Next, we screened the caecilian Hox clusters for conserved noncoding sequences, which may be indicative of potential regulatory elements. Multiple global alignments of the four caecilian Hox clusters and the homologous regions of other gnathostomes (human, wallaby, chicken, lizard, Western clawed frog, Indonesian coelacanth, spotted gar and elephant shark) were conducted using LAGAN. From these alignments, conserved noncoding elements were predicted with VISTA. This method has been shown to be effective at identifying and visualizing overtly conserved non-coding elements [37]. Because of the incompleteness of the caecilian HoxC cluster, the number of CNEs detected in the caecilian HoxC clusters may be an underestimation. Furthermore, the chicken HoxC cluster was not included in this analysis because it is currently missing many sequences (more than half of the cluster). The VISTA plots of the four Hox clusters are shown in Additional file 3.

The comparisons between caecilian and other gnathostomes identified 356 CNEs in intergenic and some intronic regions of the caecilian Hox clusters. The distribution of these CNEs is shown in Fig. 1 and Table 1. Of the four clusters, the HoxA cluster contained the highest number of CNEs, and the HoxC cluster had the highest number of CNEs in introns. The CNE densities in the 3′ part of the caecilian HoxA, HoxB and HoxD clusters (especially from HoxPG5 to HoxPG3) tended to be higher than those in the 5′ part, consistent with the previous observations in many other gnathostomes [38–41]. This density difference was not readily observed in the caecilian HoxC cluster because of the incompleteness especially in its 3′ part. Overall, CNEs tended to be located around the coding regions of the Hox genes, and the regions containing more repeats (e.g., *HoxB2*-*HoxB1*, *HoxC3*-*HoxC1*, *Evx2*-*HoxD13*, *HoxD3*-*HoxD1* and *HoxD8*-*HoxD4*) tended to have lower CNE densities.

**Table 1:** CNEs in the four Hox clusters of Banna caecilian

Cluster	Total number	Number of CNEs located in intron	Hox genes with CNEs in intron: gene name (number of CNEs)						
HoxA	109	7	*HoxA11* (2)	*HoxA10* (1)	*HoxA7* (1)	*HoxA4* (1)	*HoxA2* (2)		
HoxB	82	7	*HoxB9* (1)	*HoxB8* (1)	*HoxB7* (1)	*HoxB4* (2)	*HoxB3* (2)		
HoxC	95	10	*HoxC12* (1)	*HoxC11* (2)	*HoxC9* (2)	*HoxC8* (1)	*HoxC6* (1)	*HoxC5* (1)	*HoxC4* (2)
HoxD	70	6	*HoxD11* (1)	*HoxD10* (1)	*HoxD9* (2)	*HoxD4* (1)	*HoxD1* (1)		

For comparison, CNEs in the homologous regions of the frog Hox clusters were also identified using a similar method, and 308 CNEs were found, which was fewer than those identified in caecilian. Comparing the CNEs in the two amphibians, 251 of the caecilian CNEs (average length 144 bp) corresponded to 265 of the frog CNEs (average length 129 bp) as several long CNEs in caecilian were broken into shorter CNEs in frog. In addition to the CNEs common to caecilian and frog, 105 CNEs were identified by comparing caecilian with other non-frog gnathostomes (total length 9.1 kb), but only 43 CNEs were identified by comparing frog with other non-caecilian gnathostomes (total length 3.6 kb).

We next classified the CNEs in caecilian and frog into 4 phylogenetic groups, “gnathostome”, “osteichthyan”, “sarcopterygian” and “tetrapod”. The “gnathostome” group was defined as CNEs at the same positions in the Hox clusters of elephant shark and caecilian or frog. The “osteichthyan” group referred to CNEs shared by spotted gar and caecilian or frog. The “sarcopterygian” group referred to CNEs found in both Indonesian coelacanth and caecilian or frog. The remaining CNEs were all placed in the “tetrapod” group. Note that the CNEs already included in a previous phylogenetic group were not counted in the next groups. As shown in Table 2, a large portion of the CNEs found in both amphibians was categorized into the “tetrapod” and “gnathostome” groups. Most of the CNEs in the “gnathostome” group were conserved in almost all of the species studied and most of the CNEs in the “tetrapod” group were also found in one or two other species (Additional file 4). Because similarities in sequences from highly divergent organisms often imply functional constraints, the CNEs we identified, especially those in the “gnathostome” group, were most likely indicative of potential important functional elements. We analyzed the caecilian “gnathostome” CNEs by blast searching the corresponding human sequences against the NCBI database of human ESTs, 58 of which (length range 56–554 bp) had EST matches (minimum identity of 95 %, e-value = 10^−5^, 50 % of length overlap) and were therefore putative non-coding RNA genes. The remaining “gnathostome” CNEs likely contained many potential cis-regulatory elements; 17 of them were on the list of functionally-verified Hox enhancers summarized in a study by Ravi et al. [41]. Furthermore, CNEs in the other three groups, especially those present in multiple organisms, may also be putative non-coding RNAs or cis-regulatory elements and deserve further functional analysis.

**Table 2:** Comparing the caecilian or frog CNEs in the four phylogenetic groups

	HoxA	HoxB	HoxC	HoxD	Total
	Caecilian vs. Frog	Caecilian vs. Frog	Caecilian vs. Frog	Caecilian vs. Frog	Caecilian vs. Frog
Gnathostome	39 vs. 36	28 vs. 21	16 vs. 20	33 vs. 25	116 vs. 102
Osteichthyan	4 vs. 4	12 vs. 14	10 vs. 10	2 vs. 4	28 vs. 32
Sarcopterygian	23 vs. 9	20 vs. 0	25 vs. 14	6 vs. 5	74 vs. 28
Tetrapod	43 vs. 42	22 vs. 29	44 vs. 43	29 vs. 32	138 vs. 146
Sub total	109 vs. 91	82 vs. 64	95 vs. 87	70 vs. 66	356 vs. 308

Differences were observed between caecilian and frog in the retention of CNEs at different evolutionary stages. Among them, the most prominent were in the “sarcopterygian” group in which there was 2.6 fold greater difference in the CNEs in caecilian compared with frog (Table 2) and this difference increased to more than 5.7-fold if the CNEs common to caecilian and frog were excluded (data not shown). This difference was mostly observed in the HoxA, HoxB and HoxC clusters, especially in the HoxB cluster where caecilian had 20 “sarcopterygian” CNEs while frog had none. This variation in the “sarcopterygian” group was the primary reason for the different number of CNEs found in caecilian and frog.

### Slow evolutionary rates of caecilian genes

**Fig. 3:**
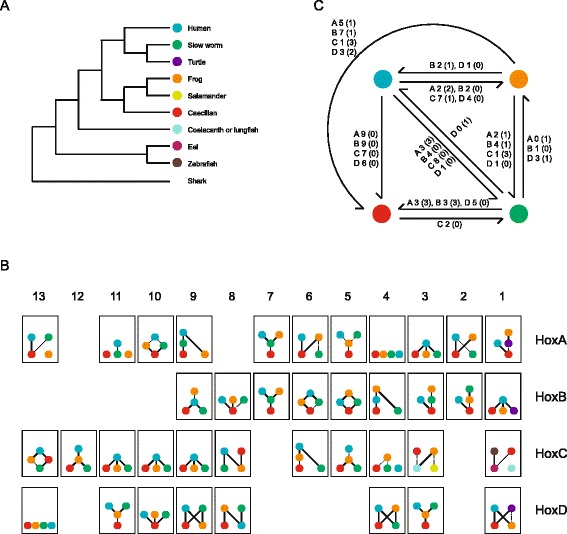
RRTs of the gnathostome Hox gene sequences. **a** Evolutionary relationship of the gnathostome species used in the RRTs of the Hox genes. **b** Summary of the RRTs conducted on the Hox gene nucleotide sequences. For most of the Hox genes, RRTs were performed using Banna caecilian, Western clawed frog, Puerto Rican worm lizard/Chinese softshell turtle and human as the ingroups and Indonesian coelacanth as the outgroup. RRTs of *HoxC1* were carried out using elephant shark as the outgroup and caecilian, African lungfish, eel and zebrafish as the ingroups to replace the species that do not have *HoxC1* gene. For *HoxC3*, the ingroups included caecilian, frog, Alpine stream salamander and African lungfish and the outgroup was Indonesian coelacanth. Results of RRTs for each gene are shown in a *Hasse diagram*, in which the slower-evolving genes are placed below the faster-evolving ones, with statistical significance denoted as a *solid line* (*p* ≤ 0.01, high significant) or a *dotted line* (0.01 < *p* ≤ 0.05, significant). **c** Summary of the significant RRTs among Banna caecilian, Western clawed frog, Puerto Rican worm lizard and human. For each pair of species, the significant RRTs are denoted by *arrows* pointing to the slower-evolving one. The number of highly significant (significant) tests for each Hox cluster is indicated on the *side* of the *arrow*

The evolutionary rates of the Banna caecilian Hox genes were estimated using the Tajima’s relative rate test (RRT) in which Banna caecilian, Western clawed frog, Puerto Rican worm lizard/Chinese softshell turtle and human were first used as the ingroups and Indonesian coelacanth as the outgroup. Because of the missing *HoxC3* and *HoxC1* in certain tetrapods, 37 of the 39 Hox genes were analyzed. As shown in Fig. 3, over 62 % of the analyzed Hox coding sequences showed significantly (*p* ≤ 0.05) slower evolutionary rates in caecilian than in frog, whereas the reverse was true for no Hox genes. Nearly 84 % of the analyzed Hox coding sequences were identified evolving significantly (*p* ≤ 0.05) more slowly in caecilian than in the mammal representative, human, whereas the reverse was true for no Hox genes. Because anole lizard’s Hox genes generally evolved fast, reptiles were represented by slow worm (our unpublished data, 34 Hox genes) and Chinese softshell turtle (3 Hox genes). Over 45 % of the analyzed Hox genes revealed significantly slower evolutionary rates in caecilian than in the reptile representatives. Thus, most of the Hox genes analyzed evolved significantly more slowly in Banna caecilian than in other species, 13 of which evolved the most slowly in Banna caecilian compared with all other species studied. Because Indonesian coelacanth Hox clusters have been reported to evolve comparatively slowly, we next compared the relative evolutionary rates of caecilian and coelacanth using the elephant shark as the outgroup. All of the Hox genes showed no significant difference in evolutionary rate, but 7 appeared to evolve faster in caecilian than in coelacanth. In summary, the Hox gene sequences evolved much more slowly in caecilian than in other tetrapod species, except for a few genes located in the HoxC cluster, and approximately 81 % of the Hox genes demonstrated similar evolutionary rates to those in the slowly evolving Indonesian coelacanth. RRTs were also performed using the Hox protein sequences (Additional file 5) to avoid inaccurate estimation due to the issue of saturation when using nucleotide sequences. Twenty one of the caecilian Hox proteins showed slower evolutionary rates than at least one other species, while only 7 frog Hox proteins were slower than at least one other species. Thirteen caecilian Hox proteins evolved more slowly than the frog ones, but only one frog Hox protein evolved more slowly than its caecilian counterpart. Thus, though the evolutionary rate differences of the Hox protein sequences were not as significant as the nucleotide sequences, caecilian still appears a slow evolving species.

**Fig. 4:**
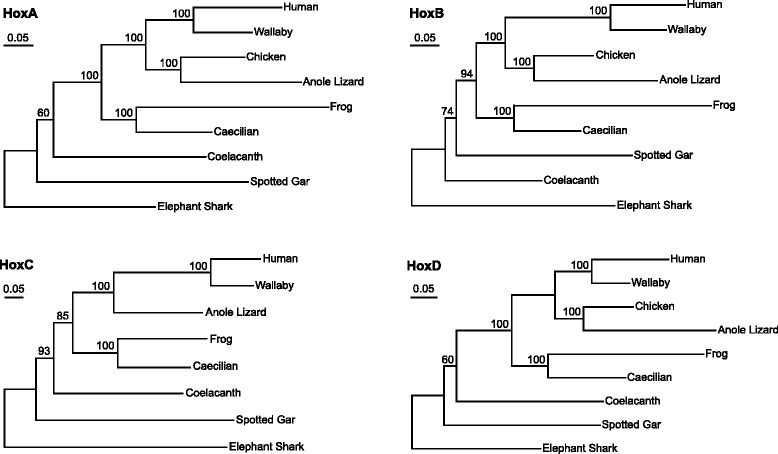
Phylogenetic trees of the gnathostome Hox gene clusters. All the alignable sequences (concatenated coding sequences and conserved noncoding sequences) for each Hox cluster were used. The trees were constructed using RAxML under the GTR + GAMMA + I model (500 rapid bootstrap replicates). Elephant shark was used as the outgroup. Values above branches denote maximum likelihood bootstrap support

Additionally, phylogenetic trees of the four Hox clusters were constructed using all of the alignable sequences (including both coding and noncoding sequences). Branch lengths reflect the differences in evolutionary rates among and within lineages with longer branch lengths representing faster evolutionary rates. As observed in Fig. 4, in the HoxA, HoxB and HoxD cluster trees branch lengths of frog were 2.6-fold (0.34 vs. 0.13), 2.9-fold (0.35 vs. 0.12) and 2-fold (0.31 vs. 0.15) longer than those of caecilian, respectively. In the HoxC cluster tree, the branch length of frog was also longer than that of caecilian, though this difference appeared to be smaller than the differences observed in the other three clusters. All of the phylogenetic trees further demonstrate that the caecilian Hox clusters evolved more slowly than the frog Hox clusters.

**Fig. 5:**
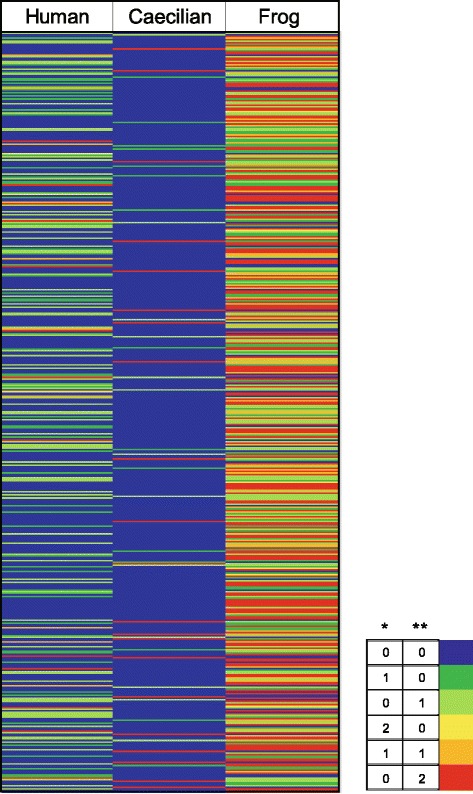
Genome-wide RRTs of Banna caecilian, Western clawed frog and human using nucleotide sequences. RRTs were performed on the nucleotide sequences of 623 orthologous genes, with Banna caecilian, Western clawed frog and human as the ingroups and Indonesian coelacanth as the outgroup. The number in a cell represents the number of statistical significance (highly significant **, significant *) for RRTs of a species compared with the others; hence, there are six possibilities for each ingroup species. Increasing *warm color* intensity indicates faster relative evolutionary rate, whereas increasing *cold color* intensity indicates slower relative evolutionary rate

Next, we wondered whether the slow evolution of the caecilian Hox clusters reflected a genome-wide phenomenon, as in the case of coelacanth [18]. To test this idea, we performed an RRT analysis with both nucleotide and protein sequences using 623 orthologous genes from human, frog and caecilian (our unpublished transcriptome data) (Fig. 5). For the nucleotide dataset, as many as 48.6 % genes in caecilian were significantly slower than the orthologs from either frog or human. Approximately 21.5 % caecilian genes showed the slowest evolutionary rate, i.e., slower than both frog and human. In frog, the percentage of genes slower than their caecilian and/or human orthologs was only 9.5 %. The RRT result of the protein sequences showed a similar trend (Additional file 6). Up to 43.5 % genes in caecilian were significantly slower than the orthologs from either frog or human. In frog, this percentage was 13.6 %. The percentages of genes showed the slowest evolutionary rate in caecilian and frog were 10.6 and 1.1 %, respectively. Additionally, we constructed a phylogenetic tree with the 623 concatenated orthologous genes from 9 vertebrate species (Additional file 7). Both nucleotide sequences and protein sequences were analyzed. In both trees, the branch lengths of caecilian were short which suggested that it evolved slowly. Among the three distinct living amphibian lineages, the evolutionary rates of frog and axolotl were both greater than that of caecilian. Thus, not only the Hox genes, but also a large portion of the protein-coding genes are evolving significantly more slowly in caecilian.

## Discussion

In this study, we successfully obtained most of the Banna caecilian Hox cluster sequences using LA PCR and GW. We identified 39 Hox genes, 2 Evx paralogs, 5 microRNA genes and 1 pseudogene (*ψHoxD12*) in four clusters. Most of their physical linkages were determined which was not accomplished in previous PCR surveys. The overall organization of the caecilian Hox clusters was conserved, similar to those from other species with four clusters. The presence of *HoxC1* in caecilian is unique compared with all the tetrapods studied so far, which suggests that the tetrapod ancestor had *HoxC1* but lost it in different lineages. Loss or pseudogenization of *HoxD12* appears to be a distinct feature of the amphibians’ Hox clusters and has been proposed to be related to digit reduction as frogs and salamanders normally only have four fingers and caecilians are limbless [20]. Other surveys have shown that *HoxD12* is also lost in snakes, which are limbless, and the African lungfish, which has only thread-like fins, but it is present in many limbless lizards [21]. Therefore, there may be different mechanisms responsible for digit reduction and *HoxD12* may not be directly involved in all of them. Alternatively, it could be the insertion of repetitive sequences that first disrupted the regulatory network of the posterior HoxD genes and altered the developmental program for fingers and even limbs. These effects of repeat insertion may then account for the pseudogenization of *HoxD12*. It would be interesting to characterize the posterior HoxD cluster in limbless lizards and see whether there are overtly multiple repeats in the region though the Hox genes remain intact.

From the obtained sequences, the caecilian Hox clusters appear compact and have fewer repetitive sequences than the Hox clusters in the other organisms used in this study. Though both are amphibians, caecilian and frog have much different repeat contents, which may reflect the different evolutionary constraints on the Hox clusters of the two amphibians. There were, however, gaps in several intergenic regions that we could not fill in caecilian. Based on the Hox cluster information from other amphibians, one possible reason is that these regions have been greatly enlarged, such as the expanded *HoxD11*-*HoxD13* in the caecilian *Typhlonectes natans* [22] and the red spotted newt [42] and the expanded *HoxC13*-*HoxC12*, *HoxC5*-*HoxC4* and *HoxD3*-*HoxD1* in the western claw frog [19]. Repetitive sequences are likely abundant in these possibly expanded regions in Banna caecilian as they have accumulated in the known large intergenic regions in the Hox clusters of many organisms [19, 22, 42]. Two of them (*HoxB2*-*HoxB1*, *HoxD13*-*HoxD11*) have actually shown signs of repeat accumulation (see Results and Fig. 1). Thus, these gapped intergenic regions were possibly repeat “hotspots” and the amount of repetitive sequences in the Banna caecilian Hox clusters may currently be underestimated. In the future, obtaining the full sequences of these gaps and determining the structural features will elucidate whether they are indeed expanded and have multiple repeat insertions. If this were the case, it would also help to understand why repeat hotspots exist. Expansion of intergenic regions may affect gene regulation by long-range regulatory elements. Insertion of repetitive DNA has the potential to alter local regulatory structures by undermining the conserved motifs and/or creating new ones, further influencing gene expression [43]. Alternatively, the presence of repetitive DNA may just indicate weakened coherence or relaxed constraint within the clusters. Why repeats are enriched in certain intergenic regions and whether they are related to developmental variations of caecilians or just a reflection of the relaxation of evolutionary constraints remains unknown.

VISTA plots demonstrated a high level of conservation in the non-protein-coding regions of the caecilian Hox clusters. In contrast to repetitive sequences, CNEs were abundant in the obtained caecilian Hox cluster sequences. And CNEs tended to be located in the regions containing few repeats. This finding may reflect the preservation of regulatory modules that formed the complex regulatory network of the Hox genes. The phylogenetic group classifications suggested that the CNEs in the Hox clusters had different conservation depths. The ancient (appeared from elephant shark) and well conserved (found in multiple vertebrate species) CNEs may be fundamental for the regulatory network and proper Hox functions. Other CNEs may then be gradually added to or removed from the regulatory network at different evolutionary stages and in different lineages, thus complicating and rewiring the regulatory network in different species. Comparative analyses of the Hox cluster sequences from more vertebrate species will improve the accuracy of this classification and elucidate the functions of the CNE. That caecilian Hox clusters contained more CNEs than frog, with the major difference in the “sarcopterygian” group, implies that the frog Hox cluster sequences evolved faster than the caecilian sequences and that the caecilian Hox cluster sequences retained more characteristics of the early tetrapods than the frog sequences, such as *HoxC1* and the *HoxD12* remnant.

The slow evolution of the caecilian Hox clusters was supported by the RRTs performed for the Hox protein sequences and the phylogenetic tree analyses of all the alignable Hox cluster sequences. The majority of the caecilian Hox genes had evolutionary rates similar to the Indonesian coelacanth Hox genes which were known to have evolved slowly. As in the case of coelacanth, the slow evolution of the caecilian Hox clusters may be indicative of its slowly evolving genome. It was supported by the RRTs and the phylogenetic tree analysis of 623 protein homologs which showed a general pattern of slow evolutionary rate of the caecilian sequences. Thus from the evolutionary rate of the Hox cluster and the 623 protein dataset, it was quite likely that the caecilian genome was evolving slowly. However, this still require support from more genomic information. For this rarely found organism, deciphering more of its genomic information and studying it from the inside out would greatly complement the studies on its morphology, physiology and function. Furthermore, to better understand the genome biology of living amphibians, a caecilian genome project is especially of practical importance, because salamanders possess genome sizes that are intractably large for routine genomic analyses [44]. And as the most basal tetrapod lineage, the caecilian genome is also highly favorable for comparative genomic studies. For example, comparative analyses between the caecilian and coelacanth genomes would provide important clues for reconstructing genomic perspectives of ancestral tetrapods and understanding the water-to-land transition. Currently, there are fewer than 1000 nucleotide sequences for caecilian in Genbank, and most of these sequences are mitochondrial gene markers from phylogenetic studies. Our study sequencing the majority of the four caecilian Hox clusters, is an important data resource and provides an amount of genomic information for the cryptic caecilian. In the future, caecilian shall be added to the list of organisms in demand of genome projects, and characterizing the caecilian genome will not only help to understand its biology, but also will provide insights into the genome biology and evolution of early tetrapods.

## Conclusions

In this study, we have cloned and sequenced most of the Banna caecilian Hox cluster. Thirty nine Hox genes, 5 microRNA genes and 1 pseudogene (*ψHoxD12*) were identified and most of their physical linkages in the four clusters were determined. The presence of *HoxC1* in caecilian suggests that the tetrapod ancestor had *HoxC1* but then lost it in different lineages. Loss of *HoxD12* function appears to be a feature of the amphibians Hox clusters. Whether and how it was related to digit reduction requires further investigation. From the obtained sequences, the caecilian Hox clusters appear compact and have fewer repetitive sequences than the Hox clusters in the other organisms studied in this work. However, to fully understand the repetitive sequences in the caecilian Hox clusters, the intergenic gaps are to be sequenced and their structural features be determined in future studies. The analysis on CNEs, the RRTs on the Hox genes and the phylogenetic trees constructed from the Hox clusters all revealed stronger constraints on the caecilian Hox clusters than their frog counterparts. These information may be indicative of a slowly evolving genome, which was supported by RRT analyses and the phylogenetic tree construction using a large orthologous protein dataset. Therefore, characterizing more of the caecilian genomic information is in demand, which will not only help to understand the basic biology of this clade, but also be highly valuable for the comparative genomics of amphibians and the evolutionary studies of early tetrapods.

## Additional files

Additional file 1:
**Global genomic alignments of the posterior HoxD cluster (from** ***HoxD13*** **to** ***HoxD11*** **).** Exons (blue boxes) and transcription direction of the genes are indicated. Sequence comparisons were conducted using the human sequence as the reference. Nucleotide identities relative to the human sequence are given by histogram peaks. CNEs are depicted by red peaks. Additional file 2:
**Global genomic alignments of part of the HoxB cluster (from** ***HoxB9*** **to** ***HoxB6*** **).** Exons (blue boxes) and transcription direction of the genes are indicated. Sequence comparisons were conducted using the human sequence as the reference. Nucleotide identities relative to the human sequence are given by histogram peaks. CNEs are depicted by red peaks. Additional file 3:
**Global genomic alignments of the four Hox clusters.** Exons (blue boxes) and transcription direction of the genes are indicated. Sequence comparisons were conducted using the caecilian Hox clusters as the reference sequence. Nucleotide identities relative to the caecilian sequences are given by histogram peaks. CNEs are depicted by red peaks. Additional file 4:
**Conservation of the CNEs in Banna caecilian Hox clusters belonging to the 4 phylogenetic groups.** Nine species were used for the global genomic alignments of the HoxA, HoxB and HoxD clusters; for the HoxC cluster, only 8 species were used due to the low sequence coverage of the chicken HoxC cluster. Numbers indicate the count of species that retained an identical CNE. From the “tetrapod” group to the “gnathostome” group, the color changes from light red to dark red gradually. Additional file 5:
**RRTs of the gnathostome Hox gene using protein sequences.** (A) Evolutionary relationship of the gnathostome species used in the RRTs of the Hox protein sequences. (B) Summary of the RRTs conducted on the Hox gene protein sequences. For most of the Hox genes, RRTs were performed using Banna caecilian, Western clawed frog, Puerto Rican worm lizard/Chinese softshell turtle and human as the ingroups and Indonesian coelacanth as the outgroup. RRTs of *HoxC1* were carried out using elephant shark as the outgroup and caecilian, African lungfish, eel and zebrafish as the ingroups to replace the species that do not have *HoxC1* gene. For *HoxC3*, the ingroups included caecilian, frog, Alpine stream salamander and African lungfish and the outgroup was Indonesian coelacanth. Results of RRTs for each gene are shown in a Hasse diagram, in which the slower-evolving genes are placed below the faster-evolving ones, with statistical significance denoted as a solid line (*p* ≤ 0.01, high significant) or a dotted line (0.01 < *p* ≤ 0.05, significant). (C) Summary of the significant RRTs among Banna caecilian, Western clawed frog, Puerto Rican worm lizard and human. For each pair of species, the significant RRTs are denoted by arrows pointing to the slower-evolving one. The number of highly significant (significant) tests for each Hox cluster is indicated on the side of the arrow. Additional file 6:
**Genome-wide RRTs of Banna caecilian, Western clawed frog and human using protein sequences.** RRTs were performed on the protein sequences of 623 orthologous genes, with Banna caecilian, Western clawed frog and human as the ingroups and Indonesian coelacanth as the outgroup. The number in a cell represents the number of statistical significance (highly significant **, significant *) for RRTs of a species compared with the others; hence, there are six possibilities for each ingroup species. Increasing warm color intensity indicates faster relative evolutionary rate, whereas increasing cold color intensity indicates slower relative evolutionary rate. Additional file 7:
**Phylogenetic tree inferred from a dataset of 623 nuclear protein-coding genes. **(A) Nucleotide tree (920,766 bp). (B) Protein tree (306,922 aa). Putative orthologous genes in two species were identified using the mutual best hit (MBH) in Basic Local Alignment Search Tool (e-value = 10^−20^). The tree was constructed using the maximum likelihood method with RAxML under the GTR + GAMMA + I model for nucleotide sequences and the LG + GAMMA model for protein sequence (500 rapid bootstrap replicates). Elephant shark was used as the outgroup. Values above branches denote maximum likelihood bootstrap support.
