# Supplementary figures and images for: Hox cluster characterization of Banna caecilian (Ichthyophis bannanicus) provides hints for slow evolution of its genome
Source: BMC Genomics. 2015 Jun 18;16(1):468. doi: 10.1186/s12864-015-1684-0 (PMC4470032; doi:10.1186/s12864-015-1684-0)

Posterior HoxD

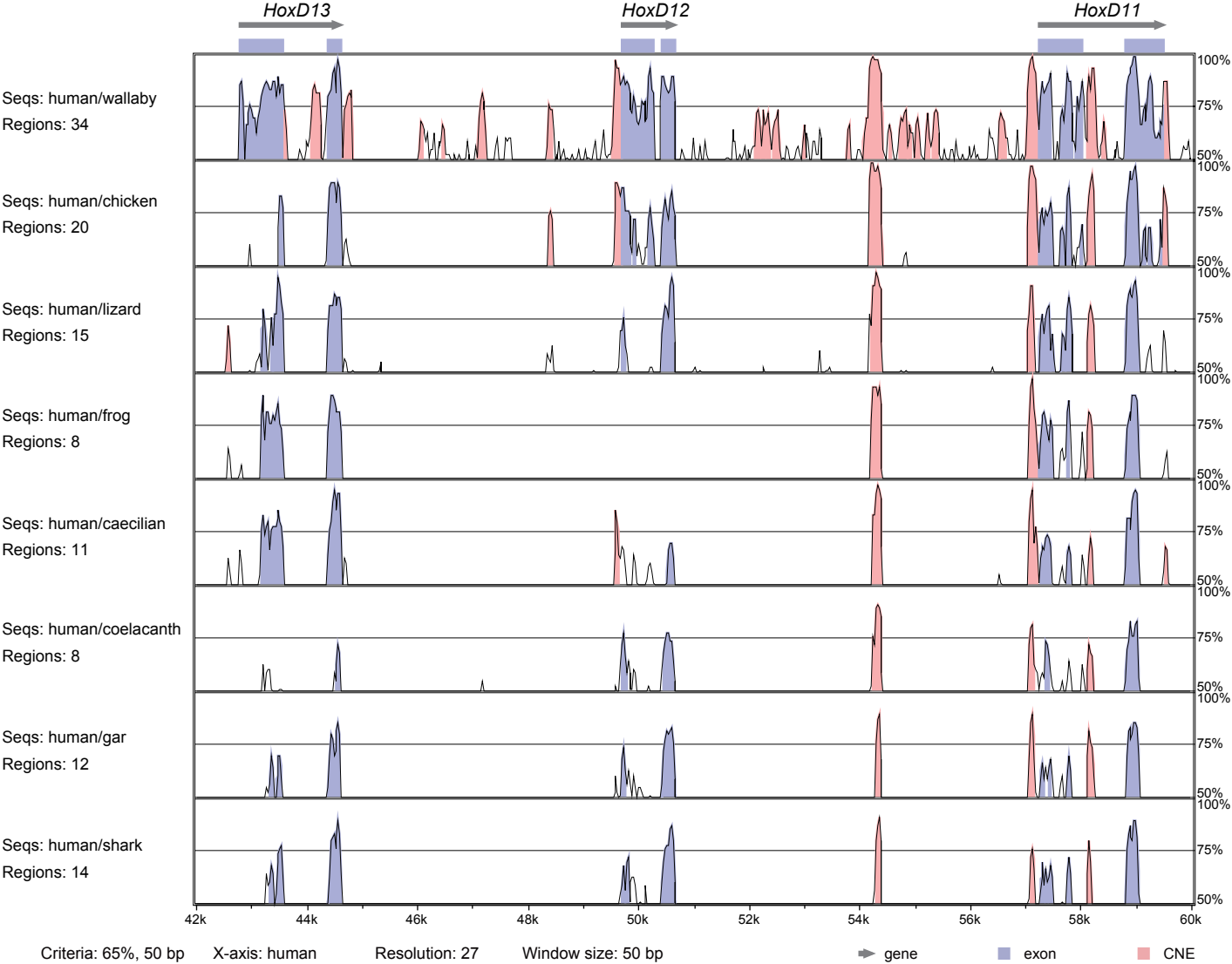

Supplement: Additional file 1: — Global genomic alignments of the posterior HoxD cluster (from HoxD13 to HoxD11 ). Exons (blue boxes) and transcription direction of the genes are indicated. Sequence comparisons were conducted using the human sequence as the reference. Nucleotide identities relative to the human sequence are given by histogram peaks. CNEs are depicted by red peaks. [file 12864_2015_1684_MOESM1_ESM.pdf]

Posterior HoxB

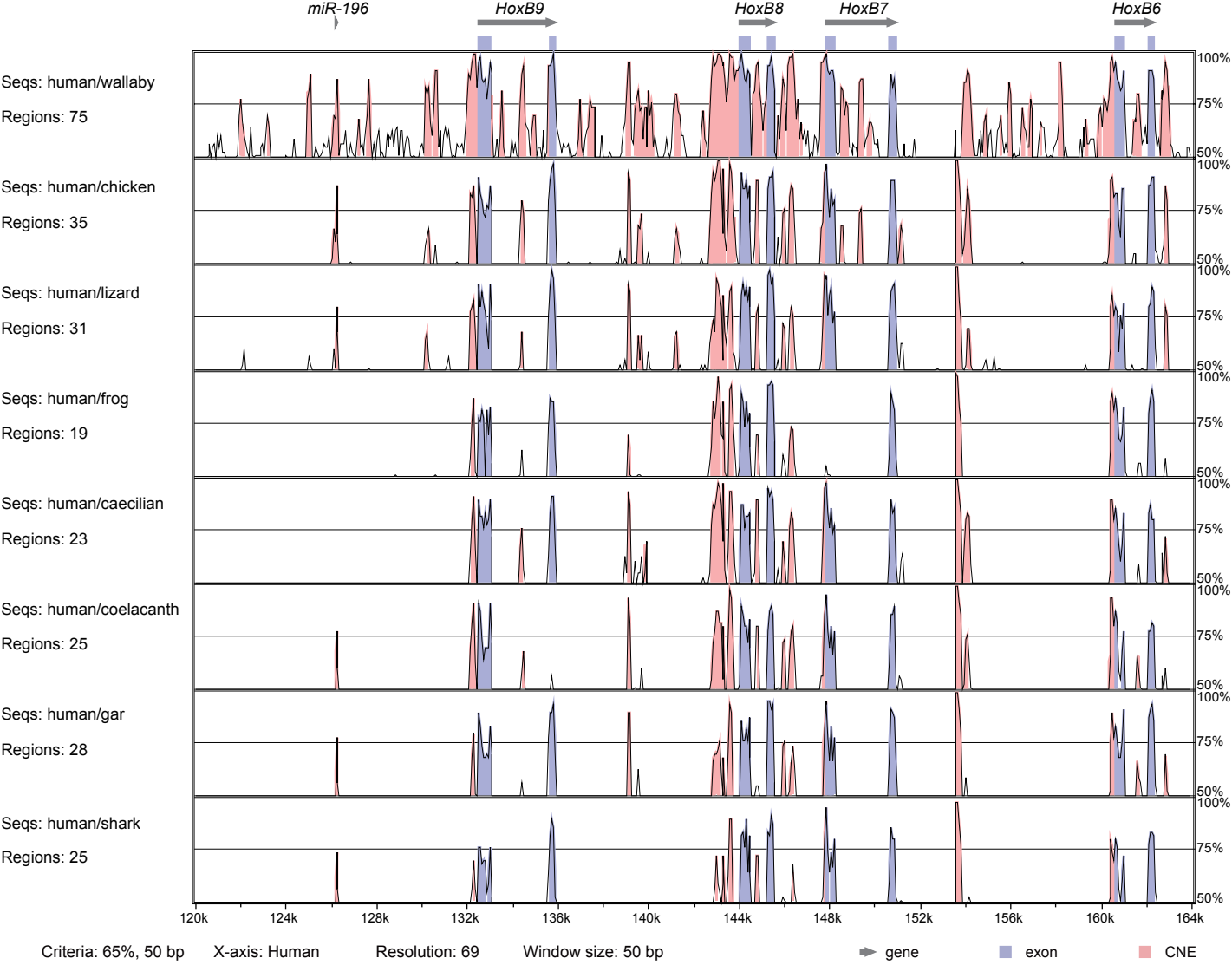

Supplement: Additional file 2: — Global genomic alignments of part of the HoxB cluster (from HoxB9 to HoxB6 ). Exons (blue boxes) and transcription direction of the genes are indicated. Sequence comparisons were conducted using the human sequence as the reference. Nucleotide identities relative to the human sequence are given by histogram peaks. CNEs are depicted by red peaks. [file 12864_2015_1684_MOESM2_ESM.pdf]

HoxA

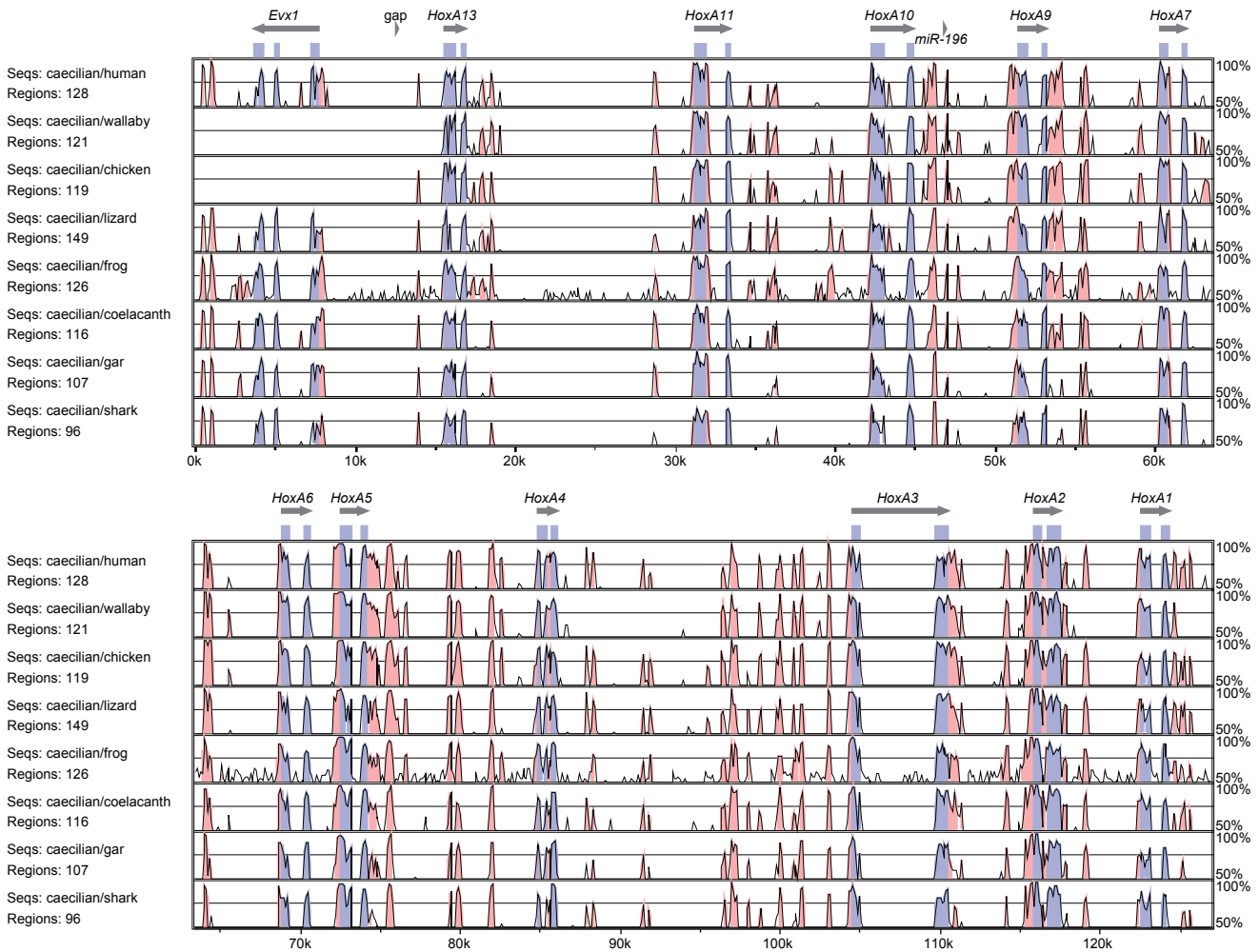

HoxB

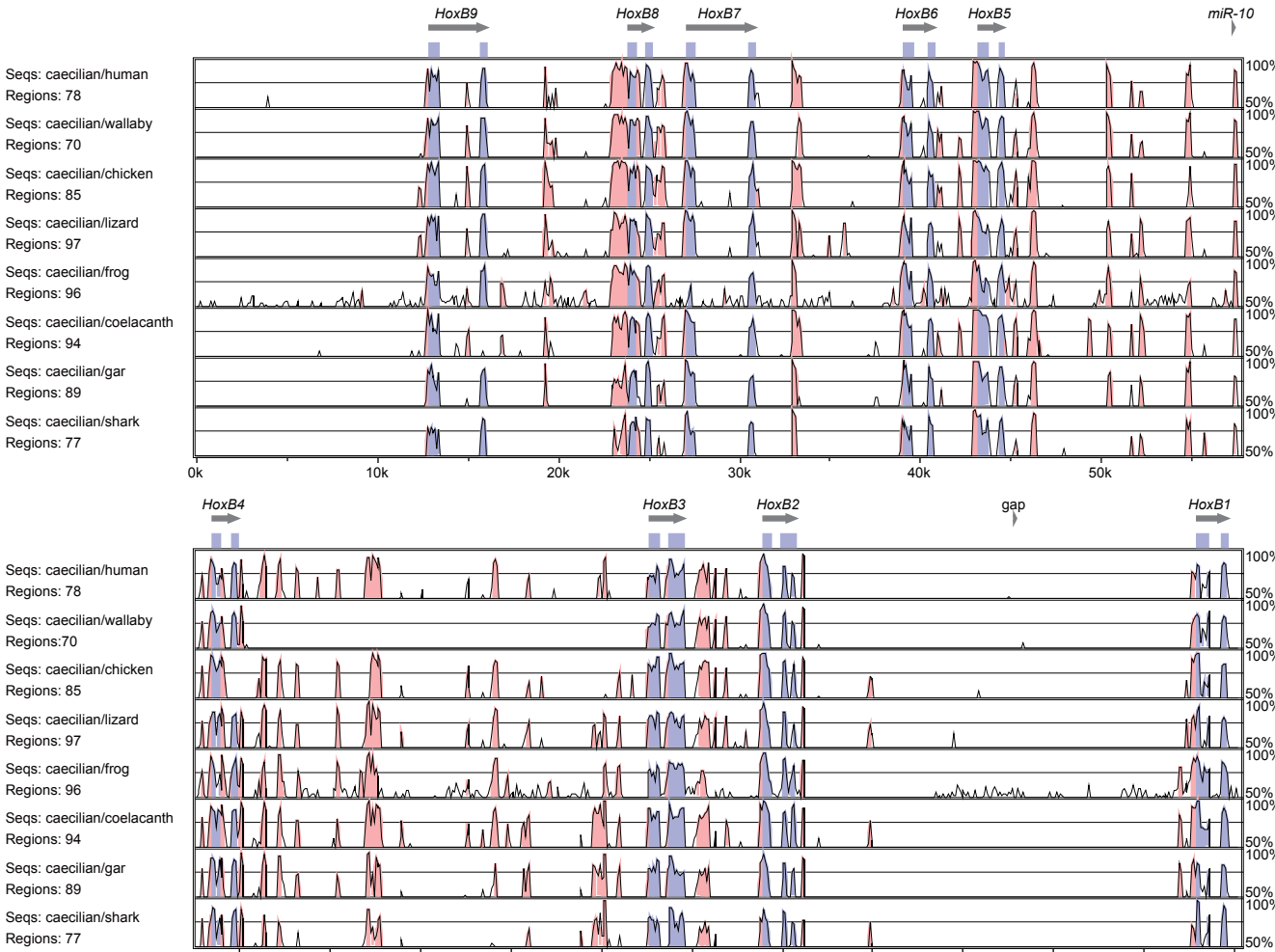

Criteria: 65%, 50 bp

X-axis: caecilian

Resolution: 91

Window size: 50 bp

➡ gene

■ exon

■ CNE

## HoxC

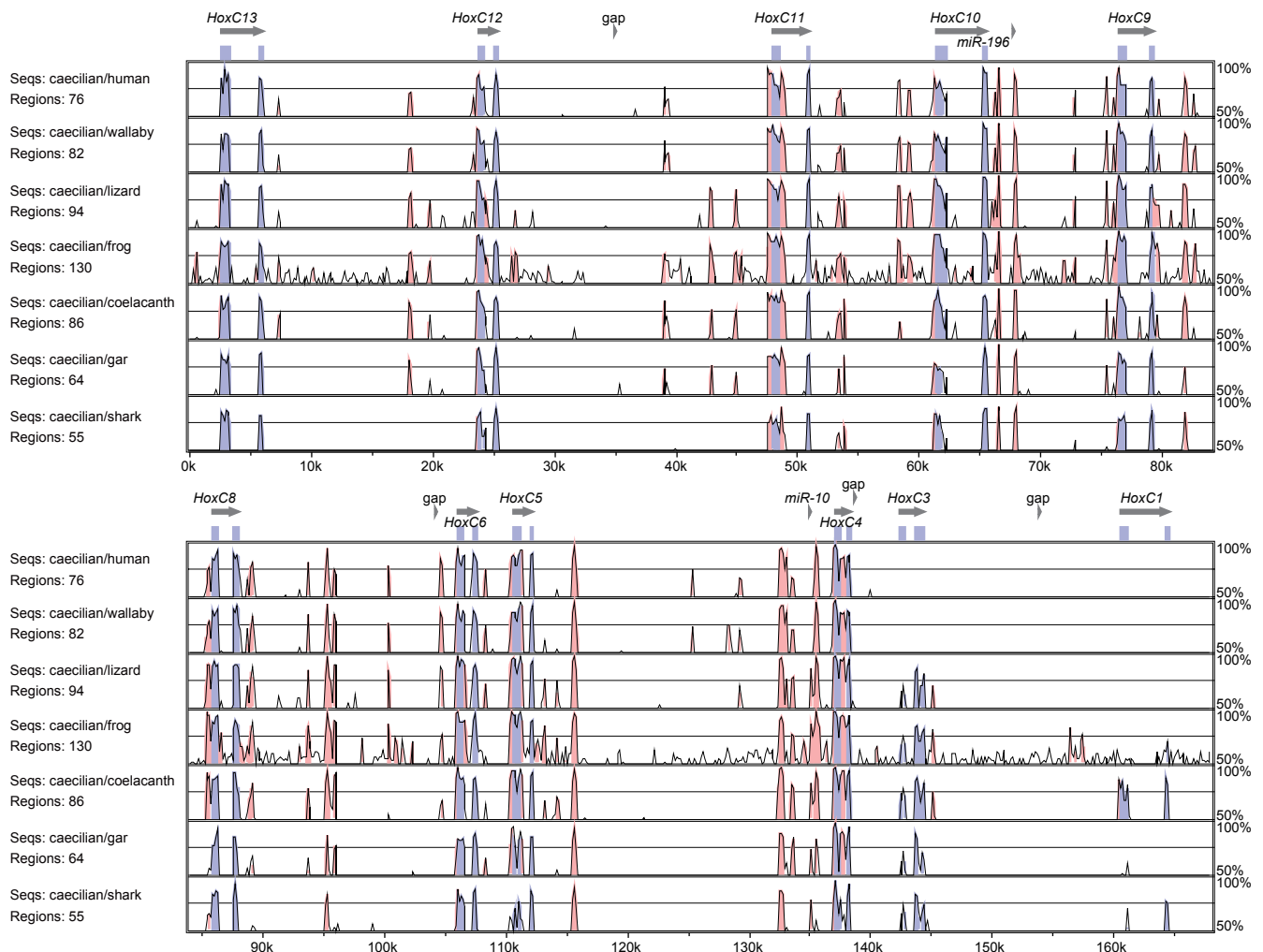

## HoxD

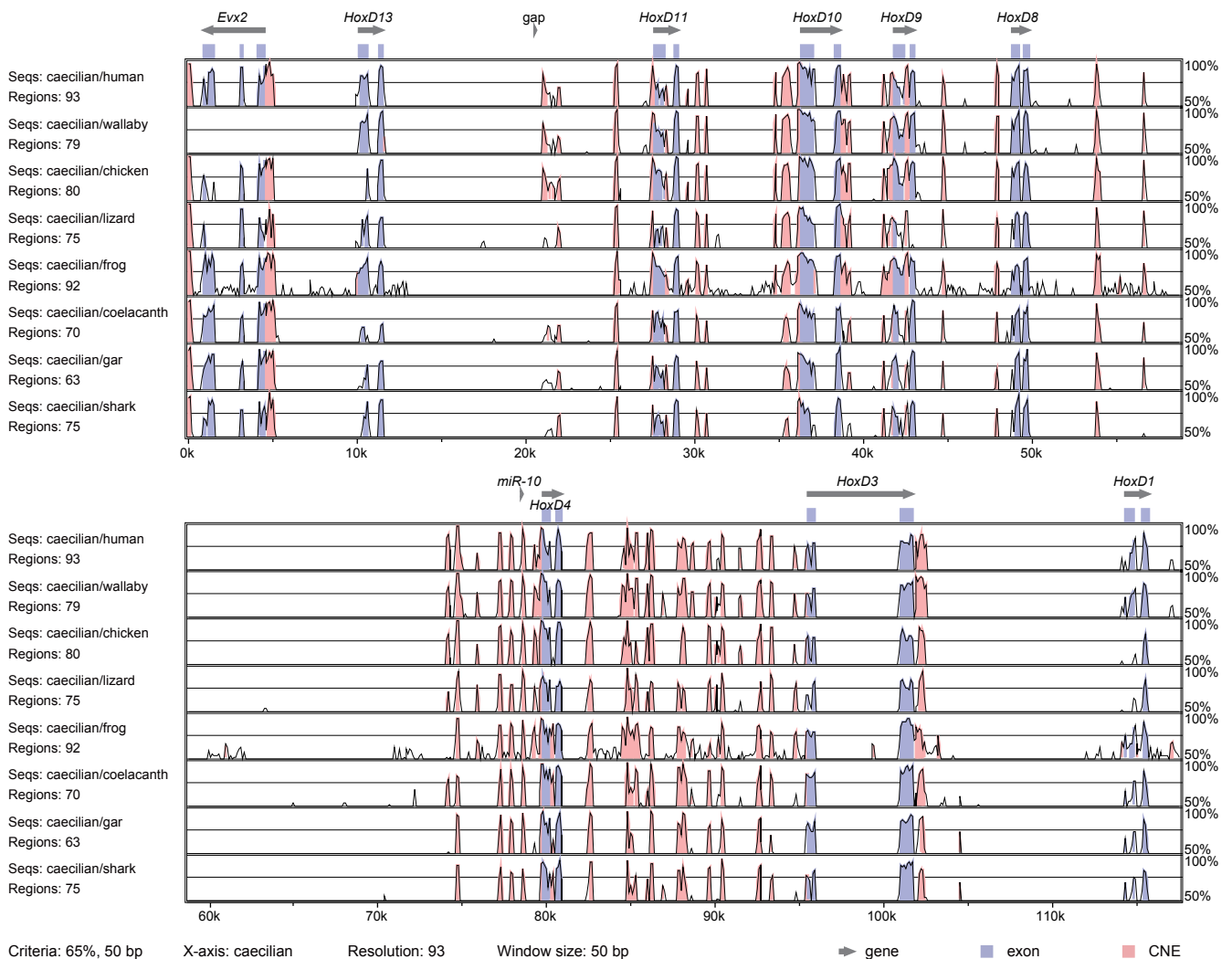

Supplement: Additional file 3: — Global genomic alignments of the four Hox clusters. Exons (blue boxes) and transcription direction of the genes are indicated. Sequence comparisons were conducted using the caecilian Hox clusters as the reference sequence. Nucleotide identities relative to the caecilian sequences are given by histogram peaks. CNEs are depicted by red peaks. [file 12864_2015_1684_MOESM3_ESM.pdf]

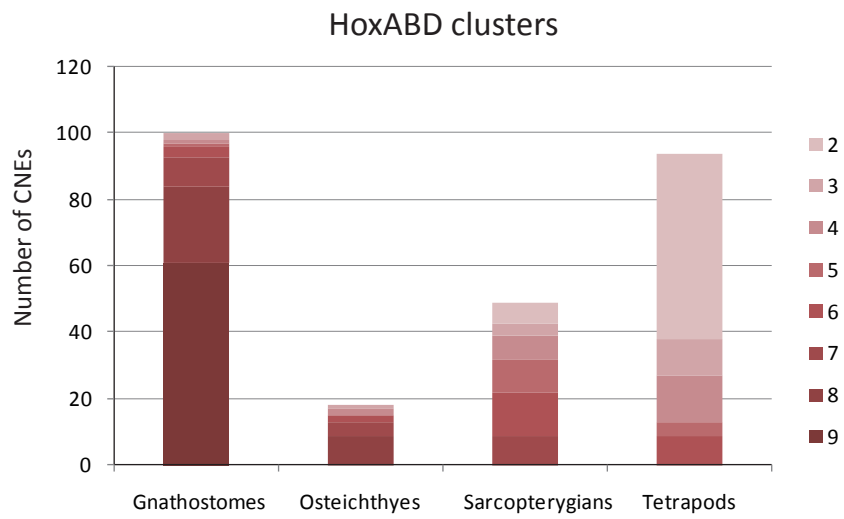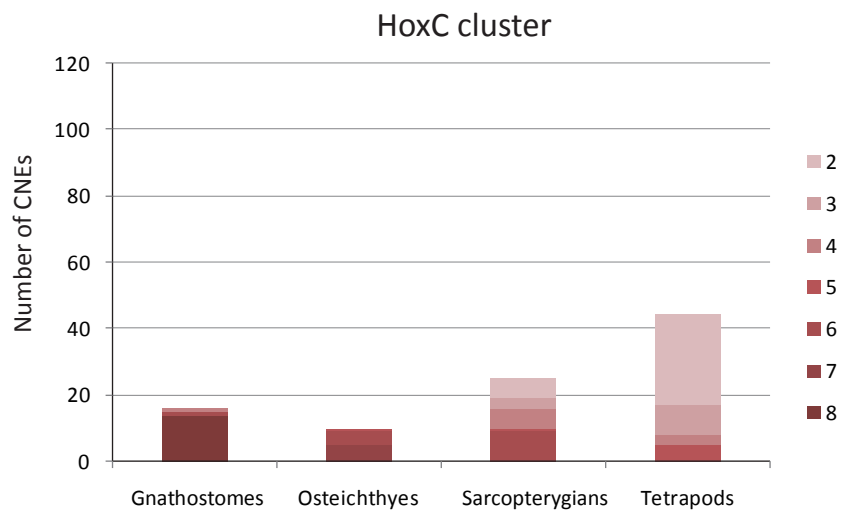

Supplement: Additional file 4: — Conservation of the CNEs in Banna caecilian Hox clusters belonging to the 4 phylogenetic groups. Nine species were used for the global genomic alignments of the HoxA, HoxB and HoxD clusters; for the HoxC cluster, only 8 species were used due to the low sequence coverage of the chicken HoxC cluster. Numbers indicate the count of species that retained an identical CNE. From the “tetrapod” group to the “gnathostome” group, the color changes from light red to dark red gradually. [file 12864_2015_1684_MOESM4_ESM.pdf]

A

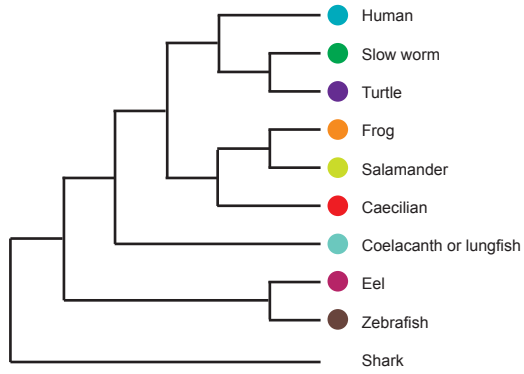

C

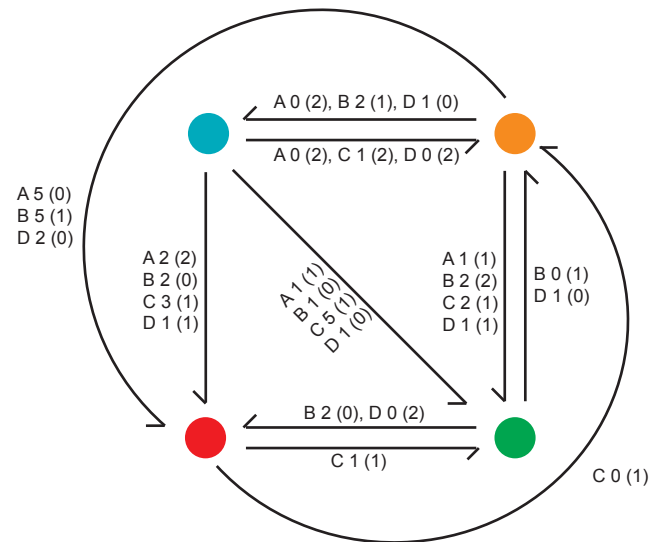

B

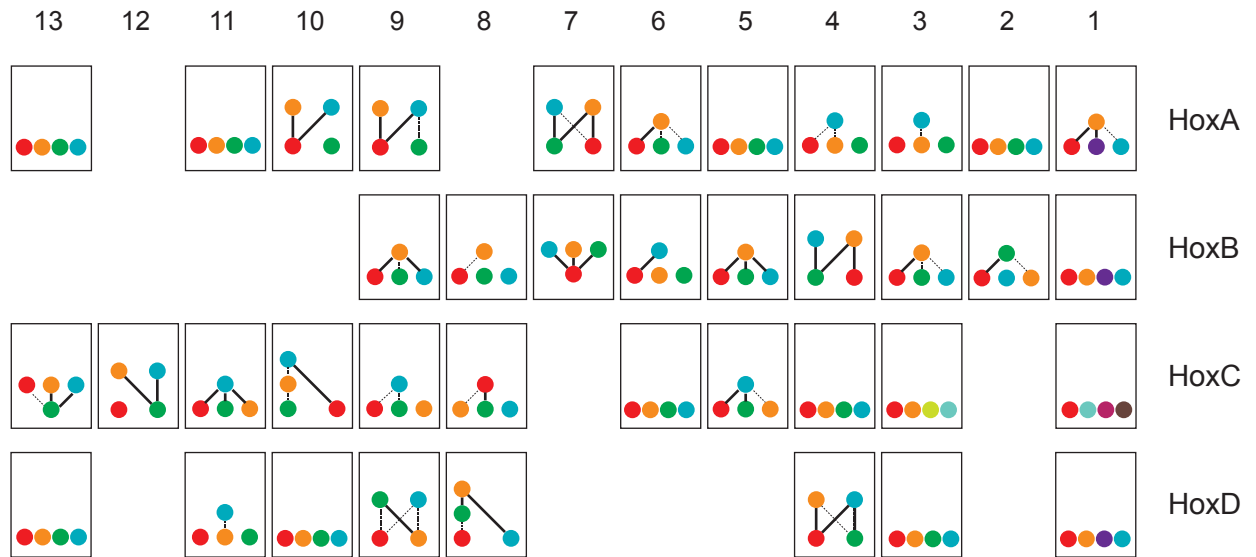

Supplement: Additional file 5: — RRTs of the gnathostome Hox gene using protein sequences. (A) Evolutionary relationship of the gnathostome species used in the RRTs of the Hox protein sequences. (B) Summary of the RRTs conducted on the Hox gene protein sequences. For most of the Hox genes, RRTs were performed using Banna caecilian, Western clawed frog, Puerto Rican worm lizard/Chinese softshell turtle and human as the ingroups and Indonesian coelacanth as the outgroup. RRTs of HoxC1 were carried out using elephant shark as the outgroup and caecilian, African lungfish, eel and zebrafish as the ingroups to replace the species that do not have HoxC1 gene. For HoxC3, the ingroups included caecilian, frog, Alpine stream salamander and African lungfish and the outgroup was Indonesian coelacanth. Results of RRTs for each gene are shown in a Hasse diagram, in which the slower-evolving genes are placed below the faster-evolving ones, with statistical significance denoted as a solid line (p ≤ 0.01, high significant) or a dotted line (0.01 < p ≤ 0.05, significant). (C) Summary of the significant RRTs among Banna caecilian, Western clawed frog, Puerto Rican worm lizard and human. For each pair of species, the significant RRTs are denoted by arrows pointing to the slower-evolving one. The number of highly significant (significant) tests for each Hox cluster is indicated on the side of the arrow. [file 12864_2015_1684_MOESM5_ESM.pdf]

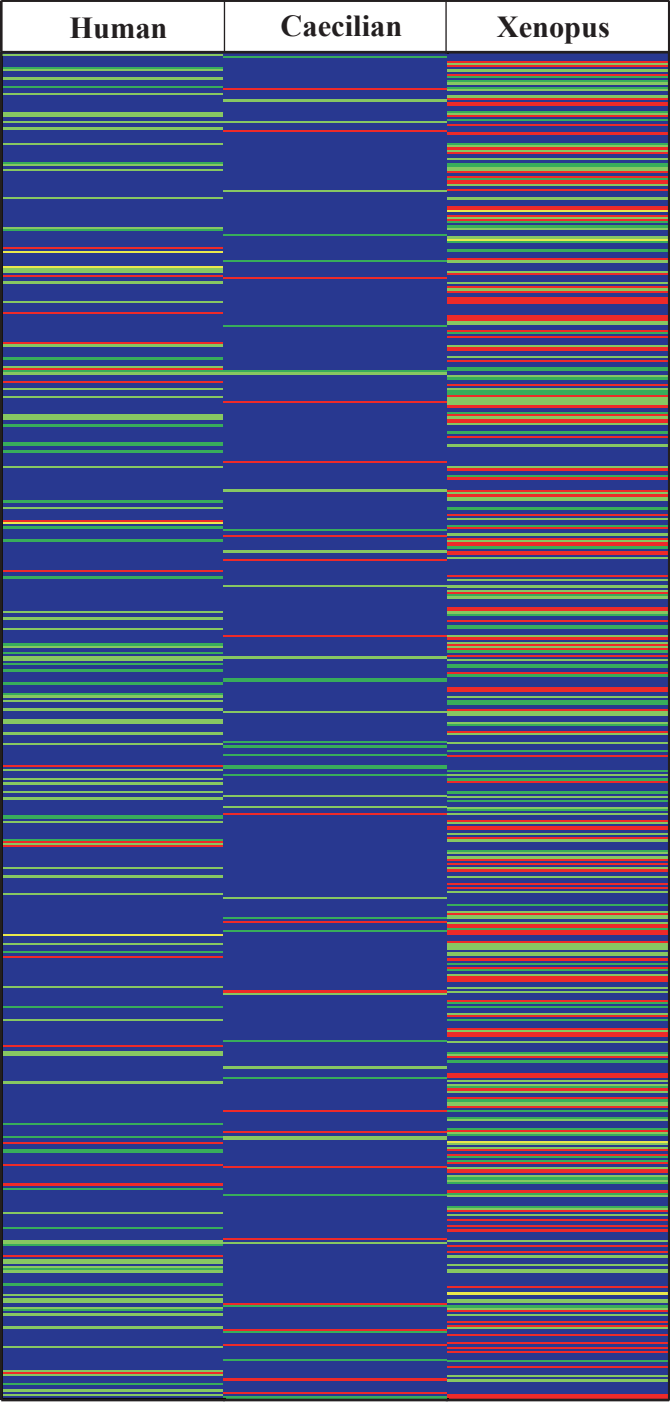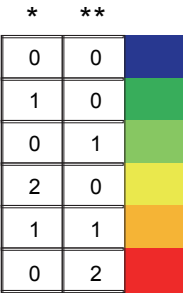

Supplement: Additional file 6: — Genome-wide RRTs of Banna caecilian, Western clawed frog and human using protein sequences. RRTs were performed on the protein sequences of 623 orthologous genes, with Banna caecilian, Western clawed frog and human as the ingroups and Indonesian coelacanth as the outgroup. The number in a cell represents the number of statistical significance (highly significant **, significant *) for RRTs of a species compared with the others; hence, there are six possibilities for each ingroup species. Increasing warm color intensity indicates faster relative evolutionary rate, whereas increasing cold color intensity indicates slower relative evolutionary rate. [file 12864_2015_1684_MOESM6_ESM.pdf]

(A) Nucleotide

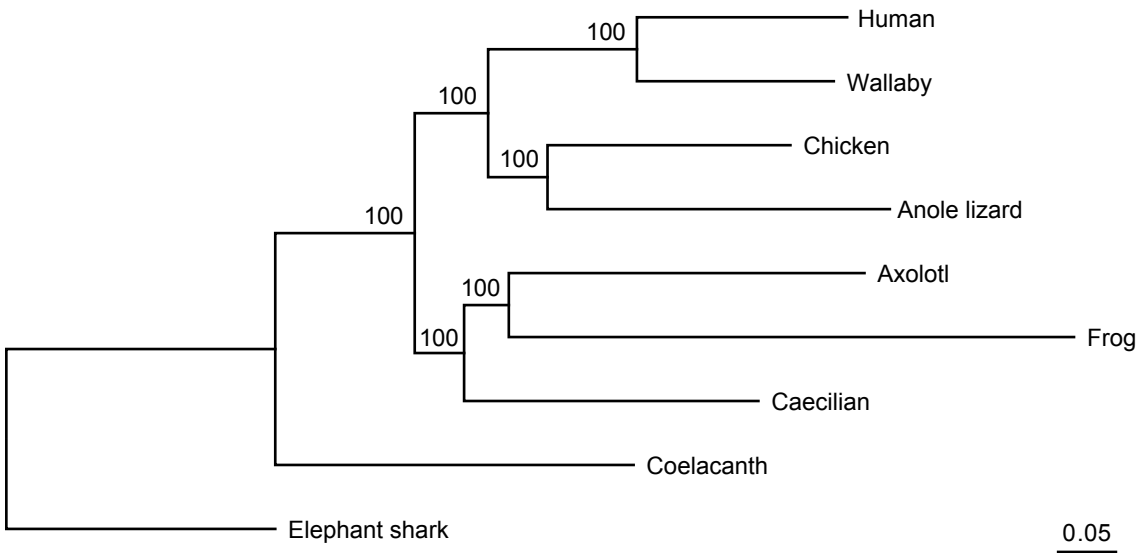

(B) Protein

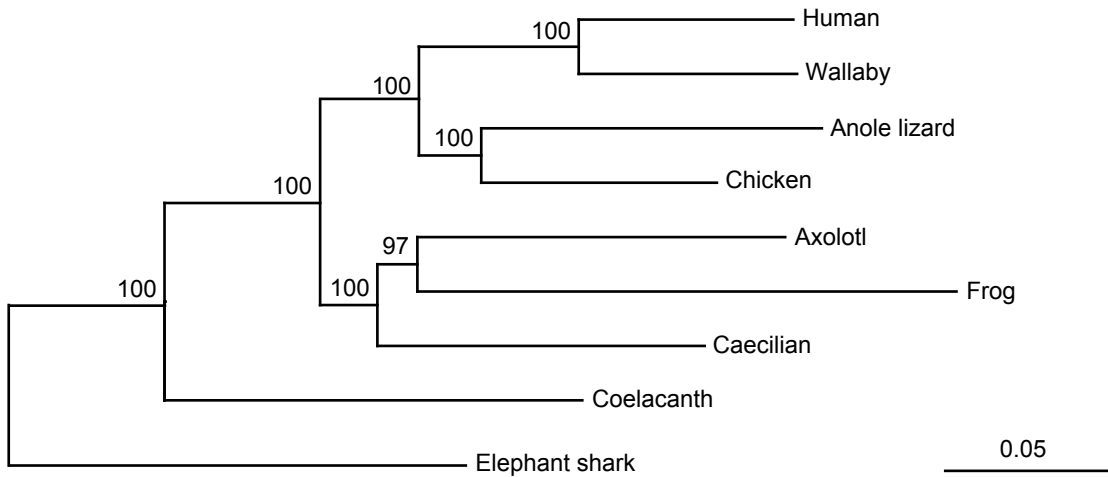

Supplement: Additional file 7: — Phylogenetic tree inferred from a dataset of 623 nuclear protein-coding genes. (A) Nucleotide tree (920,766 bp). (B) Protein tree (306,922 aa). Putative orthologous genes in two species were identified using the mutual best hit (MBH) in Basic Local Alignment Search Tool (e-value = 10−20). The tree was constructed using the maximum likelihood method with RAxML under the GTR + GAMMA + I model for nucleotide sequences and the LG + GAMMA model for protein sequence (500 rapid bootstrap replicates). Elephant shark was used as the outgroup. Values above branches denote maximum likelihood bootstrap support. [file 12864_2015_1684_MOESM7_ESM.pdf]
